# Single-cell analysis reveals cell communication triggered by macrophages associated with the reduction and exhaustion of CD8^+^ T cells in COVID-19

**DOI:** 10.1186/s12964-021-00754-7

**Published:** 2021-07-08

**Authors:** Lei He, Quan Zhang, Yue Zhang, Yixian Fan, Fahu Yuan, Songming Li

**Affiliations:** 1 Department of Blood Transfusion, Tongji Hospital, Tongji Medical College, Huazhong University of Science and Technology, Wuhan, 430030 China; 2 Department of Laboratory Medicine, Hubei Provincial Hospital of Integrated Chinese & Western Medicine, Wuhan, 430015 China; 3 Department of Physiology, School of Basic Medicine, Tongji Medical College, Huazhong University of Science and Technology, Wuhan, 430030 China; 4 School of Medicine, Jianghan University, Wuhan, 430056 China; 5 Department of Respiration, Hubei Provincial Hospital of Integrated Chinese & Western Medicine, No. 11, Linjiao Lake Road, Jianghan District, Wuhan, 430015 China

**Keywords:** COVID-19, SARS-CoV-2, Single cell RNA-sequencing, Macrophage, T cell

## Abstract

**Background:**

The coronavirus disease 2019 (COVID-19) outbreak caused by severe acute respiratory syndrome coronavirus 2 (SARS-Cov-2) has become an ongoing pandemic. Understanding the respiratory immune microenvironment which is composed of multiple cell types, together with cell communication based on ligand–receptor interactions is important for developing vaccines, probing COVID-19 pathogenesis, and improving pandemic control measures.

**Methods:**

A total of 102 consecutive hospitalized patients with confirmed COVID-19 were enrolled in this study. Clinical information, routine laboratory tests, and flow cytometry analysis data with different conditions were collected and assessed for predictive value in COVID-19 patients. Next, we analyzed public single-cell RNA-sequencing (scRNA-seq) data from bronchoalveolar lavage fluid, which offers the closest available view of immune cell heterogeneity as encountered in patients with varying severity of COVID-19. A weighting algorithm was used to calculate ligand–receptor interactions, revealing the communication potentially associated with outcomes across cell types. Finally, serum cytokines including IL6, IL1β, IL10, CXCL10, TNFα, GALECTIN-1, and IGF1 derived from patients were measured.

**Results:**

Of the 102 COVID-19 patients, 42 cases (41.2%) were categorized as severe. Multivariate logistic regression analysis demonstrated that AST, D-dimer, BUN, and WBC were considered as independent risk factors for the severity of COVID-19. T cell numbers including total T cells, CD4^+^ and CD8^+^ T cells in the severe disease group were significantly lower than those in the moderate disease group. The risk model containing the above mentioned inflammatory damage parameters, and the counts of T cells, with AUROCs ranged from 0.78 to 0.87. To investigate the molecular mechanism at the cellular level, we analyzed the published scRNA-seq data and found that macrophages displayed specific functional diversity after SARS-Cov-2 infection, and the metabolic pathway activities in the identified macrophage subtypes were influenced by hypoxia status. Importantly, we described ligand–receptor interactions that are related to COVID-19 serverity involving macrophages and T cell subsets by communication analysis.

**Conclusions:**

Our study showed that macrophages driving ligand–receptor crosstalk contributed to the reduction and exhaustion of CD8^+^ T cells. The identified crucial cytokine panel, including IL6, IL1β, IL10, CXCL10, IGF1, and GALECTIN-1, may offer the selective targets to improve the efficacy of COVID-19 therapy.

*Trial registration*: This is a retrospective observational study without a trial registration number.
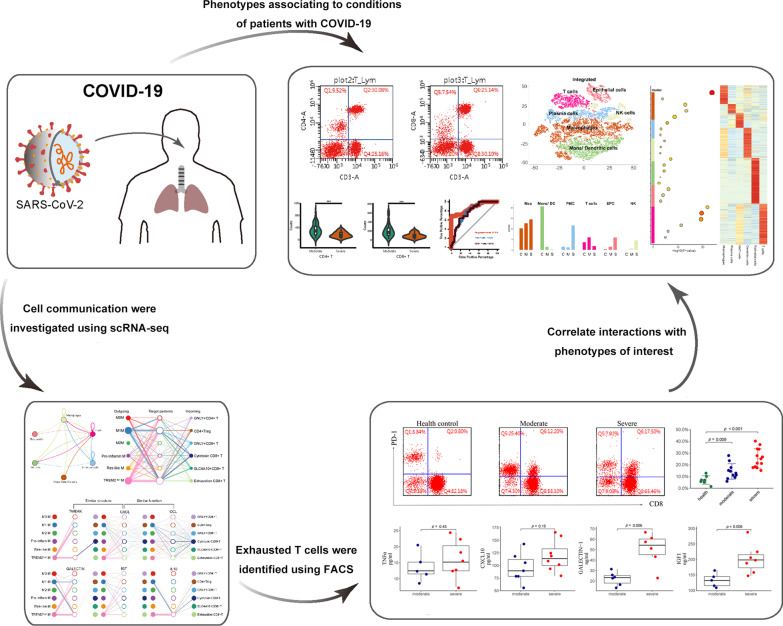

**Video Abstract**

**Supplementary Information:**

The online version contains supplementary material available at 10.1186/s12964-021-00754-7.

## Background

Coronavirus disease 2019 (COVID-19), a newly emerged respiratory disease that involves multiple organ damage, has already become an ongoing pandemic. Most patients with COVID-19 exhibit mild to moderate symptoms, but approximately 15% showing severe symptoms, and approximately 5% eventually develop acute respiratory distress syndrome with high mortality risk [1, 2]. The reasons why certain individuals are more prone to developing the severe forms of COVID-19 are also unclear. Furthermore, advanced age and comorbidities including diabetes and cardiovascular diseases, especially pathological inflammation have been observed to be the primary risk factors for severe COVID-19 symptoms [3, 4], however, the underlying immune responses at the molecular and cellular levels remain elusive.

Previous evidence suggests that SARS-CoV-2 infection and the destruction of lung cells trigger a local immune response, recruiting macrophages and monocytes that respond to the infection, release cytokines, and prime adaptive T and B cell immune responses [5]. In most individuals, the recruited cells clear the infection in the lung, the immune response recedes and patients recover. However, persistent viral infections are related to increased PD-1 expression, a marker of T cell exhaustion, leading to low circulating lymphocyte counts in severe COVID-19 cases [6]. In addition, an imbalanced immune response also triggers the development of the illness as severity does not seem to be solely related to viral load and could involve a dysregulated macrophage response associated with a cytokine storm [7, 8], and uncontrolled activation of inflammatory macrophages may trigger systemic inflammatory response syndrome (SIRS) [9, 10]. Therefore, precise regulation of cell-mediated inflammatory responses is of paramount importance in guaranteeing microbial clearance, injury limitation, and avoidance of serious side effects [11].

In the present study, we firstly retrospectively evaluated the clinical data of 102 cases of COVID-19 patients admitted to Hubei Provincial Hospital of Integrated Chinese and Western Medicine in Wuhan from February to April 2020. Consistent with the previous report [6], T cell numbers including total T cells, CD4^+^ and CD8^+^ T cells in the severe group were significantly lower than those in moderate group. To investigate potential molecular mechanisms at the cellular level using recent public single-cell RNA sequencing (scRNA-seq) data [12], we analyzed the gene expression profiles of more than 17,000 bronchoalveolar lavage fluid (BALF) immune cells from patients with COVID-19. We found that macrophages displayed specific functional diversity after SARS-Cov-2 infection, and metabolic pathway activities in the identified macrophage subtypes were influenced by hypoxia status. Importantly, we provide evidence that cell communication triggered by macrophage subtypes plays a potential roles in the reduction and exhaustion of CD8^+^ T cells.

## Methods

### Clinical data collection

We performed this retrospective study at the Hubei Provincial Hospital of Integrated Chinese and Western Medicine in Wuhan. The study involved 102 patients diagnosed with COVID-19 and hospitalized between February 3, 2020, and April 15, 2020. All cases were confirmed as COVID-19 infection by nasal and pharyngeal swab specimens and chest CT scans. The diagnosis and severity of the patients were based on the diagnosis and treatment scheme for COVID-19 (trial version 6) issued by the National Health Commission of the People’s Republic of China.

After hospitalization, the patients' serum specimens were collected for laboratory examination including flow cytometric analysis. Once the endpoints (discharge or death) were reached, we collected patient clinical data. In this study, epidemiological, clinical, laboratory, and radiological characteristics and treatments, as well as outcome data were obtained from electronic medical records. Data collection forms were reviewed independently by two researchers.

### Laboratory examination

Throat-swab specimens from the upper respiratory tract that were obtained from patients at admission were maintained in the viral transport medium. SARS-CoV-2 was confirmed by using TaqMan One-Step RT-qPCR Kits from Shanghai Huirui Biotechnology Co. Ltd and Shanghai BioGerm Medical Biotechnology Co. Ltd, both of which have been approved by the China Food and Drug Administration. The specific PCR primers, probes, and the determination of results for the detection of SARS-CoV-2 followed the recommendations of the National Institute for Viral Disease Control and Prevention, Chinese Center for Disease Control and Prevention.

Routine blood tests were performed using an XS-1000i hematology analyzer (Sysemx, Japan). Biochemical indicators were tested using the ADVIA2400 chemistry system (Siemens, Germany). Coagulation tests were performed using an ACLTOP750 automatic coagulation analyzer (Instrumentation Laboratory, USA).

Flow cytometry analysis (FACs) was performed using a BriCyte E6 flow cytometer (Mindray, Shenzhen, China). Briefly, heparinized peripheral blood was collected from study participants. After collection, Fluorescently-labeled monoclonal antibodys (Abs) were added to label the surface markers of stimulated or unstimulated cells. To label intracellular antigens, the cells were fixed and permeabilized with Fixation/Permeabilization Buffer (BD Biosciences) and then stained with Abs derived from FACS test kit (Mindray) as follows: anti-CD3-FITC (clone: SK7), anti-CD4-APC (clone: SK3), anti-CD8-PE (clone: SK), and anti-CD45-PerCP (clone: 2D1). In addition, anti–PD-1 was purchased from (BD Biosciences, catalog 557860). Isotype ontrols with irrelevant specificities were included as negative controls. The percentages and absolute numbers of total T cells, CD4^+^ T cells, and CD8^+^ T cells were determined by using the the microfluidic detector of BriCyte E6.

### Single-cell RNA-seq data preprocessing

The scRNA-seq data for most of the analyses in this study were downloaded from the GEO database (GSE145926). These data were published by Liao et al*.* [12], wherein BALF cells were extracted from nine COVID-19 patients and three healthy controls. We excluded low-quality cells based on two quality measures: the number of aligned reads < 2e5 or the number of genes detected < 200. In the subsequent analysis, we downsampled cells and normalized the gene expression profiles to reduce the influence of technical noise as previously published study [13].

### Functional and pathway enrichment analysis

Normalized gene expression data were used for pathway analysis. Gene Ontology (GO) and Gene Set Enrichment Analysis (GSEA) using the ClusterProfiler [14] or Gene Set Variation Analysis (GSVA) [15] R packages were performed on differentially expressed genes (DEGs). GO terms were identified with a strict cutoff of P < 0.01 and a false discovery rate (FDR) < 0.05.

### Single-cell trajectory reconstruction and analysis

Single-cell pseudotime trajectories were constructed using MONOCLE (version 2.6.4) [16]. Briefly, we first selected a set of ordering genes that showed differential expression between the clusters. Then, Monocle then uses reversed graph embedding, a machine learning technique to generate a parsimonious principal graph, and then reduces the given high-dimensional expression profiles to a low-dimensional space. Single cells were projected onto this space and ordered into a trajectory with branch points. As called in Monocle, cells in the same segment of the trajectory have the same ‘state’. Branched expression analysis modeling was used to further test for branch-dependent gene expression.

### Classification of hypoxia status across different samples

We selected a 15 gene expression signatures (ACOT7, ADM, ALDOA, CDKN3, ENO1, LDHA, MIF, MRPS17, NDRG1, P4HA1, PGAM1, SLC2A1, TPI1, TUBB6, and VEGFA) that has been shown to perform optimally when classifying hypoxia status in the previous study [17]. Hypoxia scores for each cell were calculated by using GSVA based on 15 mRNA-based hypoxia signatures. The Wilcoxon rank-sum test was used to assess the statistical difference with hypoxic status in different cell subpopulations.

### Ligand–receptor networks

First, to represent cell–cell communication networks via ligand–receptor interactions, we implemented a directed, weighted network as previously described [18]. Briefly, we initially considered all ligand–receptor pairs expressed in at least 10% of the cells in a population. We then built a network using six subpopulations identified in the BALF immune (BAI) cells dataset. To filter out downregulated ligand–receptor connections, we set a minimum path weight of 1.5. An overall weight describing the strength of the connection between a source and target population, the total weight can then be calculated by summing all path weights between the source and target. Finally, we further re-verified cell–cell communications among all macrophage and T cell sub-populations using the CellChat R package [19].

### Quantification of cytokine serum levels

The serum levels of cytokines, including IL6, IL10, IL1β, GALECTIN-1, IGF1, CXCL10, and TNFα, were quantified using the IMMULITE® 2000 immunoassay system (Siemens, Los Angeles, California, USA) according to the manufacturer’s instructions.

### Statistical analyses

All statistical analyses were performed using R (3.6.2). Continuous variables are expressed as mean ± standard deviation (SD). Categorical variables are summarized as the counts and percentages (%). χ2 test was performed to compare the frequencies to categorical variables. Fisher’s exact test was used to evaluate permutation-based differential proportion analysis (DPA). The differences of Categorical variables between two groups were analyzed using Student's t test (parametric) and Wilcoxon rank-sum test (non-parametric)*.* The univariate and multivariate logistic regression analyses were performed to evaluate risk factors for the disease. Statistical significance was defined as p value < 0.05.

## Results

### Laboratory characteristics of patients with moderate and severe COVID-19

From February to April 2020, 102 moderately to severely ill patients with confirmed COVID-19 were transferred or admitted to the Hubei Provincial Hospital of Integrated Chinese and Western Medicine. Of these patients, the mean age was 63.8 years, 44 (43.1%) were women and 58 (56.9%) were men. Fever (89.1%), cough (72.3%), fatigue (84.5%), anorexia (46.46%) and diarrhea (22.1%) were the most common symptoms.

According to China’s National Health Commission Guidelines for COVID-19 Treatment (6^th^ edition) published by the National Health Commission of China, 60 (58.8%) cases were classified into the moderate group, and 42 cases (41.2%) were categorized into the severe group. As shown in Table 1, white blood cell count (WBC), neutrophil %, neutrophil to lymphocyte ratio (NLR), aspartate aminotransferase (AST), total bilirubin (TBIL), blood urea nitrogen (BUN), C-reactive protein (CRP), prothrombin time (PT), and D-dimer levels were significantly higher in the severe cases than in the moderate cases (p < 0.05). Inversely, lymphocyte % and platelet counts were significantly lower in the severe group than in the moderate group (p < 0.05). These laboratory findings concurred with those of several previous studies reported [20, 21].

**Table 1:** Lab characteristics

Characteristic	Moderate (n = 60) (Mean ± SD)	Severe (n = 42) (Mean ± SD)	P value
*Age (year)*			
≤ 65	38	14	0.005
> 65	22	28	
*Gender*			
Female	27	17	0.802
Male	33	25	
WBC (10^9^/L)	6.55 ± 2.82	9.15 ± 5.95	0.011
Neutrophil %	71.51 ± 12.6	82.15 ± 10.98	< 0.001
Lymphocyte %	20.31 ± 10.72	13.27 ± 14.21	0.008
Neu/Lym	8.21 ± 2.8	22.5 ± 5.32	< 0.001
RBC (10^12^/L)	4.07 ± 0.67	4.12 ± 0.73	0.708
HB (g/L)	124 ± 19.2	125.31 ± 21.37	0.752
PLT (10^9^/L)	249.7 ± 89.47	196.26 ± 100.61	0.007
ALT (U/L)	29.38 ± 32.33	46.93 ± 52.79	0.060
AST (U/L)	27.27 ± 13.61	63.55 ± 64.47	0.001
TBIL (*u*mol/L)	12.95 ± 6.75	18.27 ± 13	0.018
BUN (mmol/L)	5.51 ± 3.27	10.34 ± 8.79	0.001
Cr (*u*mol/L)	84.6 ± 129.76	121.17 ± 138.32	0.181
CK (U/L)	119.14 ± 145.11	342.92 ± 581.34	0.024
CRP (mg/L)	26.49 ± 37.28	42.11 ± 35.22	0.044
PCT (ng/mL)	0.89 ± 6.12	5.63 ± 18.19	0.119
BNP (pg/mL)	105.27 ± 200.91	359.35 ± 100.5	0.123
PT (seconds)	12.48 ± 1.75	13.48 ± 1.73	0.006
APTT (seconds)	30.05 ± 3.46	30.63 ± 4.26	0.468
D-dimer (*u*g/mL)	1.34 ± 2.25	13.24 ± 17	< 0.001
FIB (g/L)	3.31 ± 1.09	3.55 ± 1.49	0.365

*p* values comparing severe cases and moderate cases are from χ2, or unpaired 2-sided Student’s t test

**Fig. 1:**
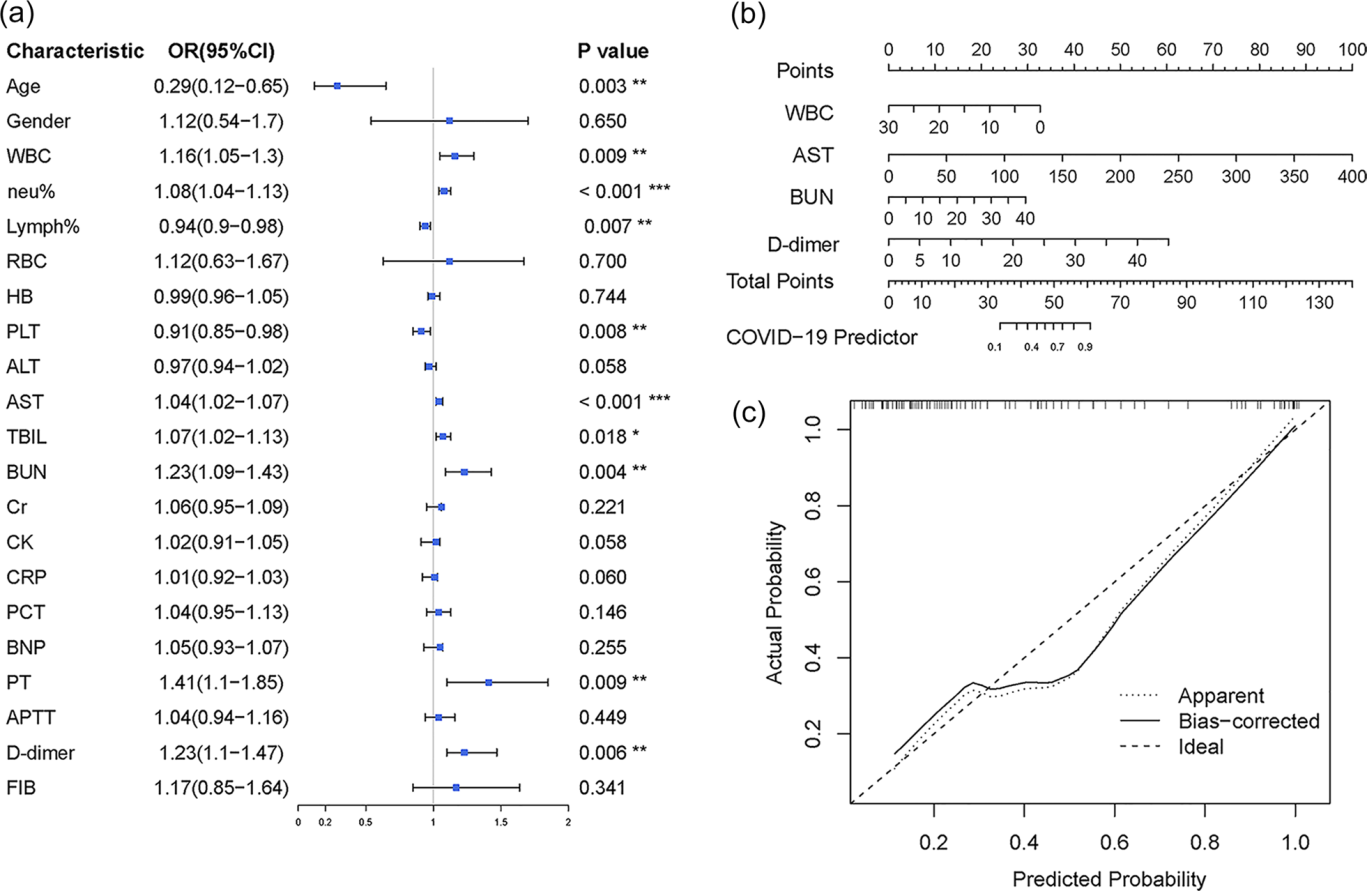
Logistic regression model performance and clinical usefulness of selected laboratory characteristics nomogram. **a** Forest plot showing the odds ratio of clinical parameters analyzed by univariate logistic regression in patients with moderate or severe COVID-19. The length of the horizontal line represents the 95% confidence interval for each indicator. The vertical dotted line represents the odds ratio (OR) = 1, OR > 1.0 implies a positive relationship. (i.e. odds ratio for gender had been above 1, this means that being male would correspond with higher odds of being the severe outcome.) **b** The nomogram was built by the multivariate logistic regression model, with the laboratory characteristics including WBC, AST, BUN, and D-dimer. **c** Calibration curve with Hosmer–Lemeshow test of the nomogram. The calibration curve depicts the calibration of the fitted model in terms of the agreement between the predicted risk of severe COVID-19 and real observed outcomes

Thereafter, we performed univariate and multivariate logistic regression analyses to evaluate risk factors for the progression of COVID-19. A forest plot (Fig. 1a) showed that a significant association between COVID-19 progression and age (≤ 65 vs. > 65 years, odds ratio (OR), 0.29), WBC count (OR, 1.16), neutrophil % (OR, 1.08), lymphocyte % (OR, 0.94), PLT (OR, 0.91), AST (OR, 1.04), TBIL (OR, 1.07), BUN (OR, 1.23), PT (OR, 1.41) and D-dimer (OR, 1.23). Multivariate logistic regression analysis demonstrated that AST, D-dimer, BUN, and WBC were considered as independent predictive variables for COVID-19 deterioration (Additional file 1: Table S1). As shown in Fig. 1b, c, the regression model including these four variables was suitable for predicting COVID-19 patient deterioration.

### CD4^+^ and CD8^+^ T cell numbers were extremely despressed with the severity of COVID-19 patients

**Fig. 2:**
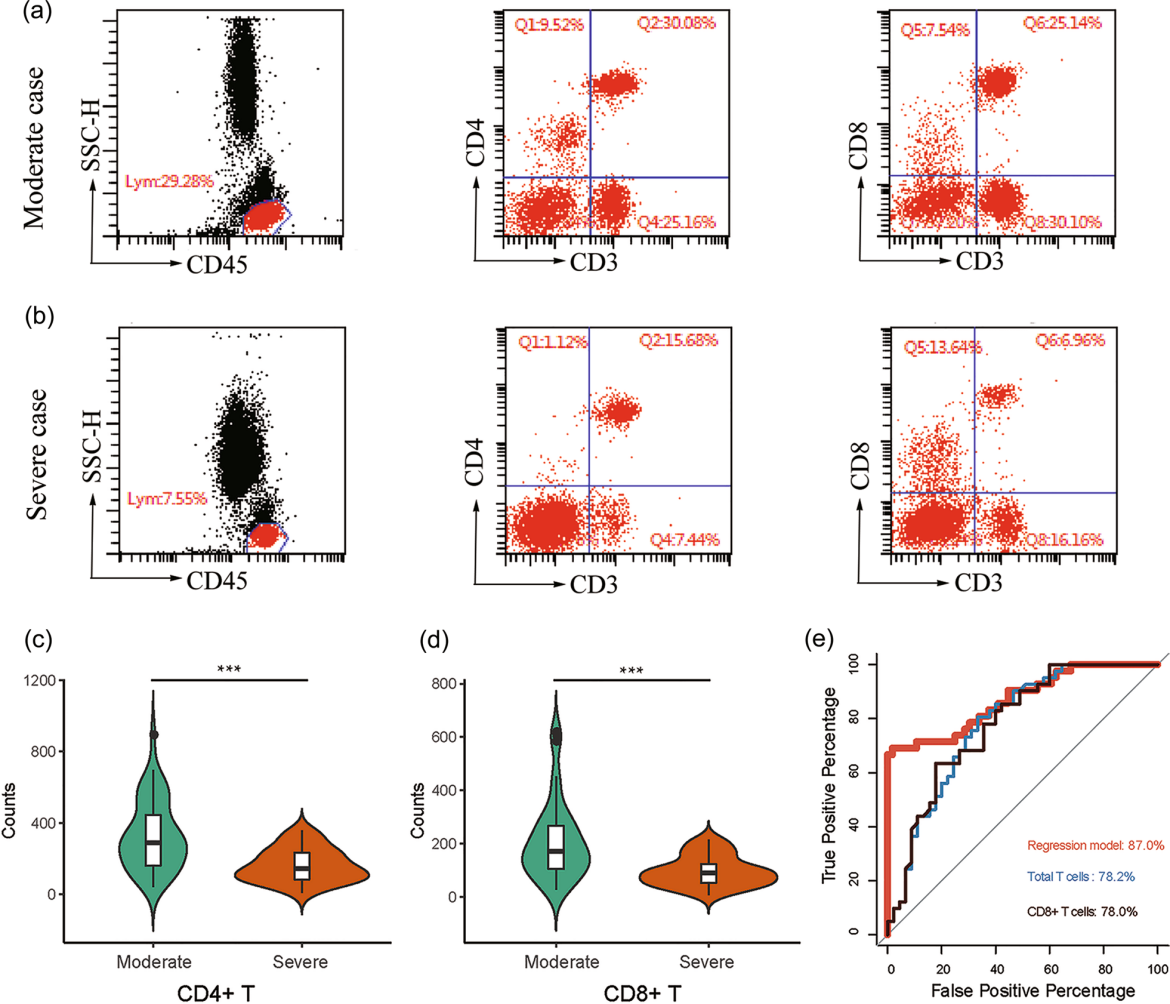
The phenotypes and counts of T cell subtypes. **a**, **b** Fluorescence-activated cell sorting (FACS) dot plot examples, gated on total CD45^+^ cells (left), the expression of CD4 and/ or CD3 on CD45^+^ cells (middle), and the expression of CD8 and/ or CD3 on CD45^+^ cells (right). Case A indicates one of the moderate patients with COVID-19 (**a**). Case B, one representative case of severe patients (**b**). **c**, **d** The violin graph showing the counts of CD4^+^ T cells (c) and CD8^+^ T cells (**d**) in patients with different groups. *** *p* < 0.001 (Student’s t-test). **e** ROCs are created by plotting the true positive rate (TPR) against the false positive rate (FPR) at various threshold settings with corresponding AUCs labeled around the curves

T lymphocytes, especially CD8^+^ cytotoxic T cells, are the most important immune cells that protect against viral infection [22]. In line with this, we next detected total T lymphocyte counts and CD4^+^ and CD8^+^ T cell counts by FACS. The absolute numbers of total T lymphocytes, CD4^+^ T cells, and CD8^+^ T cells in severe patients were all significantly lower than those in patients with moderate disease (Fig. 2a–d). The mean values of total T lymphocytes, CD4^+^ and CD8^+^ T cell counts were 521 (SD = 188), 330 (SD = 194), and 209 (SD = 147), respectively in moderate cases, and the mean value decreased to 273 (SD = 142), 163 (SD = 93), and 96 (SD = 57), respectively in severe cases. Next, we determined area under the receiver operating characteristic curve criterion (AUROC) to calculate the sensitivity and specificity of the multivariate logistic regression model, total counts of T cell, CD8^+^ T cells, and CD4^+^ T cells. The ROC curve indicates the probability that the model predicted for a randomly chosen positive case will exceed the result for a randomly chosen negative case (Fig. 2e), which indicates that the absolute numbers of total T cells and CD8^+^ T cells indicated good prognostic prediction efficacy for COVID-19 severity.

### Single-cell RNA-seq of total COVID-19 BALF cell population

The number of total T cells and CD4^+^ and CD8^+^ T cells was dramatically reduced in COVID-19 patients, especially, which was strongly correlated with patient deterioration. To further elucidate the molecular mechanisms of the above-mentioned clinical features, we analyzed transcriptome profiles at the single-cell level, which is one of the first available datasets to demonstrate the landscape of BALF immune (BAI) cells in patients with COVID-19 [12]. The dataset was selected because it covered three patients with moderate COVID-19, six patients with severe infection, and three healthy controls. Furthermore, the samples derived from BALF contained microenvironment information regarding bronchioles and lung alveoli.

**Fig. 3:**
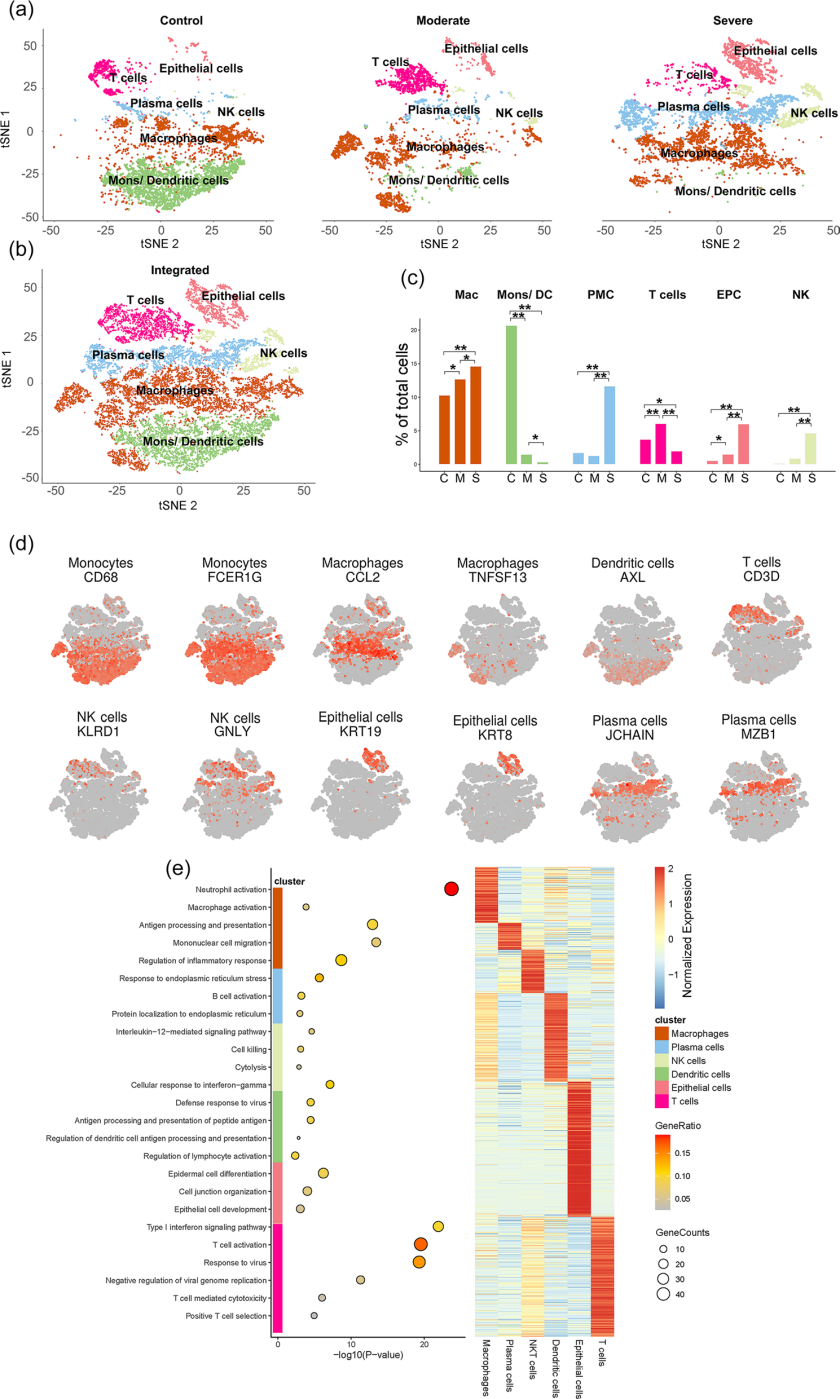
Single-cell RNA-seq of total bronchoalveolar lavage fluid immune (BAI) cells population. **a** t-SNE plots showing detected lineages and sub-populations in BAI cells across conditions (e.g. Control means the cells derived from healthy donors). **b** t-SNE plot of aggregate BAI cells with identified sub-populations. **c** Cell population percentages across conditions determined to be significantly modulated according to Differential proportion Analysis (DPA). *p < 0.05, **p < 0.01 (Fisher’s exact test). Abbreviation: C, M, S indicate Control, Moderate, and Severe, respectively; Mac, Macrophage; Mons /DC, Monocytes/Dendritic cells; PMC, Plasma cells; EPC, Epithelial cells; NK, natural kill cells. **d** Expression of select marker genes across BAI cells as visualized on t-SNE plots. **e** Left: bubble plot showing representative GO terms according to cell types. Right: heatmap characterizing the expression signatures of top 50 specifically expressed genes in each cell type; the value for each gene is row-scaled Z score

Here, we first applied data downsampling and normalization to gene expression profiles to reduce the influence of technical noise. Transcriptional profiles of 17778 cells were captured after quality control filtering (control: 6583; moderate: 4233; severe: 6962) (Fig. 3a, b, and Additional file 2: Figure S1). BAI cells were identified by a total of six distinct major cell lineages. The cell types comprised macrophages (Mac; CD68^+^CCL2^+^), monocytes/dendritic cells (Mons/DC; FCER1G^+^AXL^+^), T cells (CD3D^+^), natural killer cells (NK; KLRD1^−^GNLY^+^), plasma cells (JCHAIN^+^MZB1^+^) and epithelial cells (EPCs; KRT8^+^KRT19^+^) (Fig. 3d). Next, we performed the DPA [18] to analyze whether changes in the proportion of populations were greater than expected by chance alone. DPA identified six subtypes of BAI cells showing significant (p < 0.05) differences compared to the control samples. As shown in Fig. 3c, T cell numbers decreased markedly in the proportion of severe samples compared to control and moderate samples, whereas NK cells were expanded in severe samples, the results of which were in accordance with FACS data. To further validate the function of these cell clusters, we mapped the gene expression profiles of well-defined cell-type-specific markers in the subtypes of BAI cells, and then analyzed the biological function of each cell cluster by using Gene Ontology (GO) analysis of differentially expressed genes (DEGs) (Fig. 3e), revealing the unique characteristics of these BAI immune cells. For example, GO terms specific to macrophages included ‘‘macrophage activation,’’ ‘‘antigen processing and presentation,’’ and “Mononuclear cell migration”. GO terms including ‘‘type I interferon signaling,’’ “T cell activation” and ‘‘response to virus,’’ were enriched for T cells. Collectively, we identified six different BAI immune cell types and annotated the biological functions of each.

### scRNA-seq analysis highlights macrophages heterogeneity in responding to SARS-CoV-2 infection

**Fig. 4:**
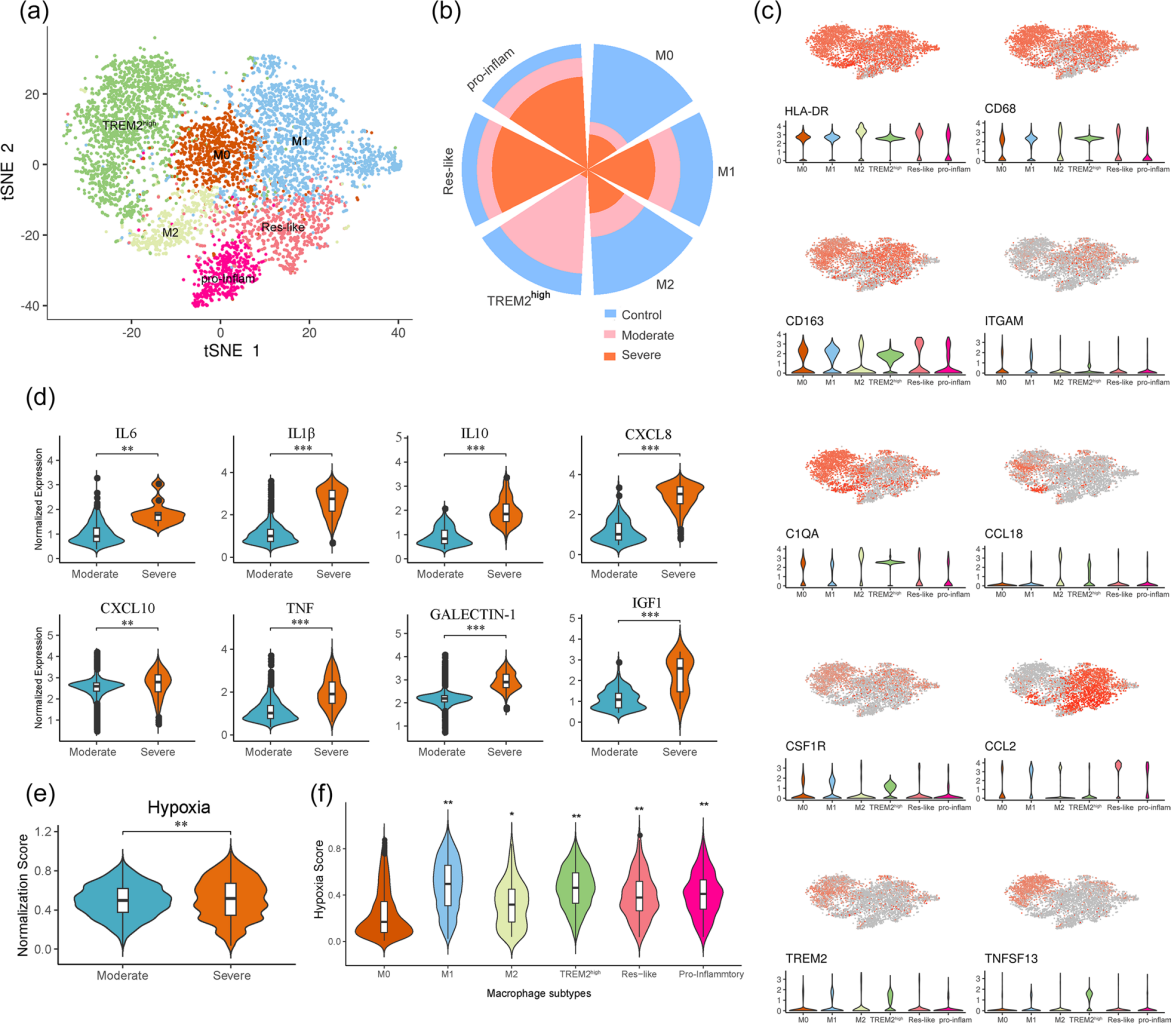
BAI macrophage subpopulations. **a** t-SNE plot showing macrophage subpopulations. **b** Percentage of the subpopulation of macrophages derived from patients with COVID-19 across different conditions. **c** Expression of marker genes across macrophage subtypes as visualized on t-SNE (upper) and violin plots. **d** The difference of cytokine expression levels between moderate and severe groups. **e** Hypoxia scores of macrophages derived from moderate and severe samples. **f** Hypoxia score enriched in each of macrophage subpopulation, the score of subpopulations were respectively compared to M0. * *p* < 0.05, ** *p* < 0.01, *** *p* < 0.001 (Student’s t test)

It is known that inflammatory macrophages play a crucial role in phagocytosis and microbial clearance; however, uncontrolled activation of inflammatory macrophages may trigger systemic inflammatory response syndrome (SIRS) or even sepsis [9, 23]. Accordingly, we next focused on exploring the transcriptional heterogeneity of macrophages in responding to SARS-Cov-2 infection. In this study, we showed that enriched populations of Res-like Mac (HLA-DR^+^ITGAM^−^CD68^low^), M1 Mac (CD68^+^ITGAM^+^CCL2^+^), and pro-inflammatory Mac (CD68^+^ITGAM^−^CSF1R^−^CCL2^+^) in patients with severe COVID-19 (Fig. 4a–c). The prominent M2 Mac (C1QA^+^CCL18^+^), and M0 Mac (CD68^+^CD163^low^ITGAM^+^) were enriched in normal controls compared to COVID-19 patients (Additional file 2: Figure S2a). Particularly about TREM2^high^, which showed the greatest percentage elevation in moderate group (Fig. 4b), also overexpressed genes involving complement activation and immune cell differentiation, including C1QA, CSF1R, and TNFSF13 (Fig. 4c). Next, We further observed that severe COVID-19 patients showed upregulation of cytokines and chemokines compared to moderate ones, suggesting the functional diversity between each of the macrophage subpopulations analyzed. For example, proinflammatory cytokines including IL6, IL1β, and TNF are thought to be produced by M1 Mac, whereas M2 Mac can secrete anti-inflammatory cytokines, such as IL10. Furthermore, previous evidence indicated that differential modulation of the chemokines including CXCL8 and CXCL10, integrates polarized macrophages in pathways of resistance to microbial pathogens (Fig. 4d). Importantly, several cytokines are expressed in a TREM2-dependent manner in macrophages. Among these, genes such as GALENTIN-1 could be a modulator of inflammatory response involving T cell exclusion [24, 25]. In accordance with the clinical symptoms of patients with COVID-19, the hypoxia gene signature was enriched in the severe groups compared to the moderate cohort (Fig. 4e). Indeed, based on this 15-gene signature, polarized macrophages exhibited significantly higher hypoxia scores than M0 (Fig. 4f), suggesting that functional heterogeneity between identified macrophage subtypes may be influenced by hypoxic status.

**Fig. 5:**
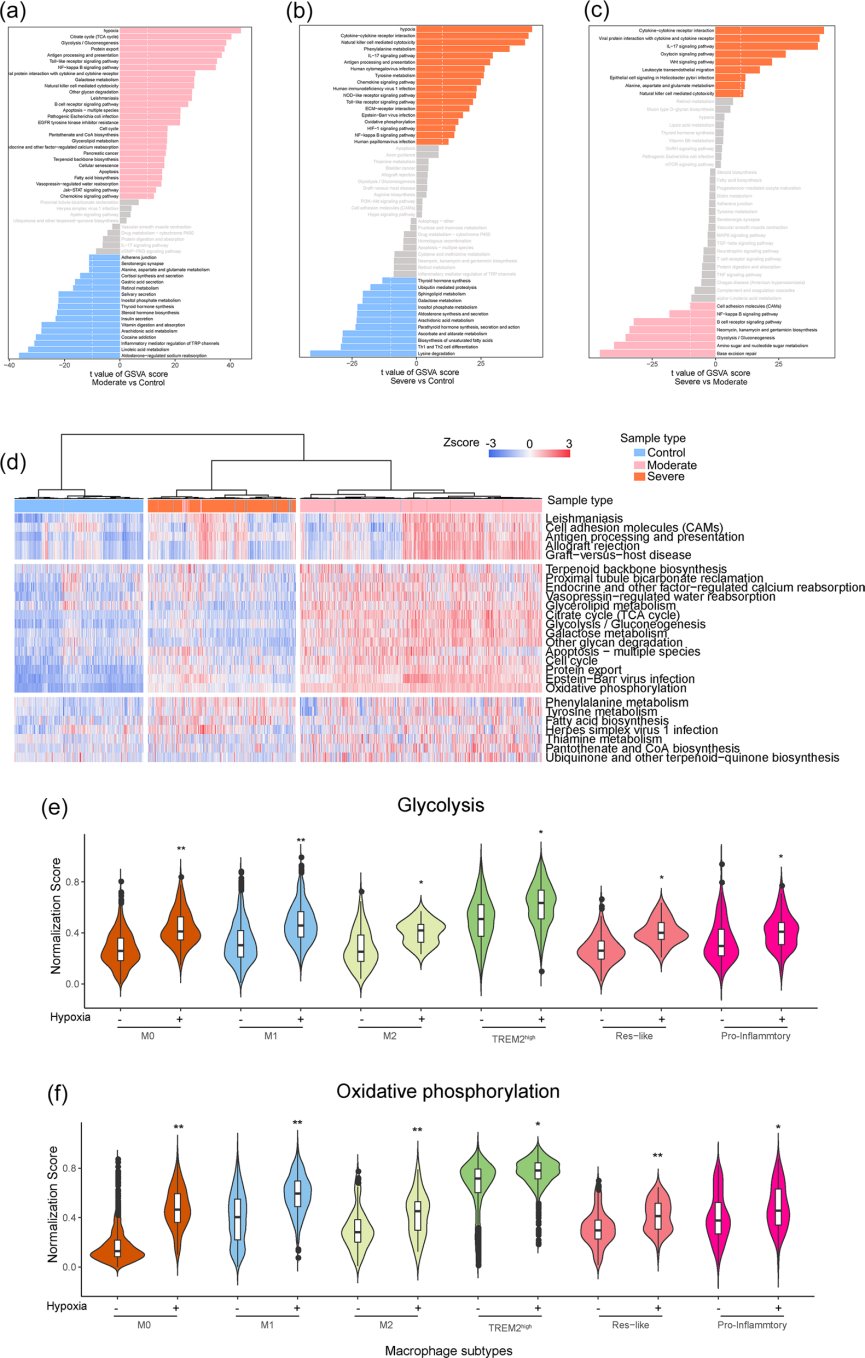
Functional heterogeneity of macrophage subpopulations after SARS-CoV-2 infection. **a**–**c** Differences in pathway activities scored per macrophages by GSVA with Moderate vs. Control (**a**), Severe vs. Control (**b**), Severe vs. Moderate (**c**). **d** Heatmap of GSVA score of top 25 signal pathway for each macrophage across conditions. the value for each GSVA score is row-scaled. **e**, **f** Signal pathway activity of glycolysis (**e**) and oxidative phosphorylation (**f**) under hypoxia status across macrophage subtypes. * *p* < 0.05, ** *p* < 0.01 (Student’s t test)

Next, we performed enrichment analysis of hallmark pathway activities scored per cell by GSVA, and conducted direct comparisons of the control group versus the moderate or severe groups, respectively. We found that the signal activity of inflammatory responses, cytokine receptor interactions, and antigen processing, and presentation were enriched in macrophages derived from COVID-19 patients, which are the hallmarks of the M1 and pro-inflammatory Mac described in previous studies (Fig. 5a–c) [26–28]. Unsupervised hierarchical cluster analysis was based on the normalized activity scores of the 30 representative hallmark pathways, and macrophage populations were separated into three groups, importantly, which almost matched to the control, moderate and severe types of patients (Fig. 5d). This result suggested that macrophages display specific functional diversity after SARS-CoV-2 infection. Importantly, the activity of metabolic pathways across macrophage subtypes was influenced by hypoxia status (Additional file 2: Figure S2b). For example, glycolysis and oxidative phosphorylation were determined by cell type-specific manner (Fig. 5e, f), suggesting that the metabolism of macrophages is more sensitive to environmental factors, especially hypoxia.

### Pseudo-time trajectory reconstruction with macrophage subpopulation

**Fig. 6:**
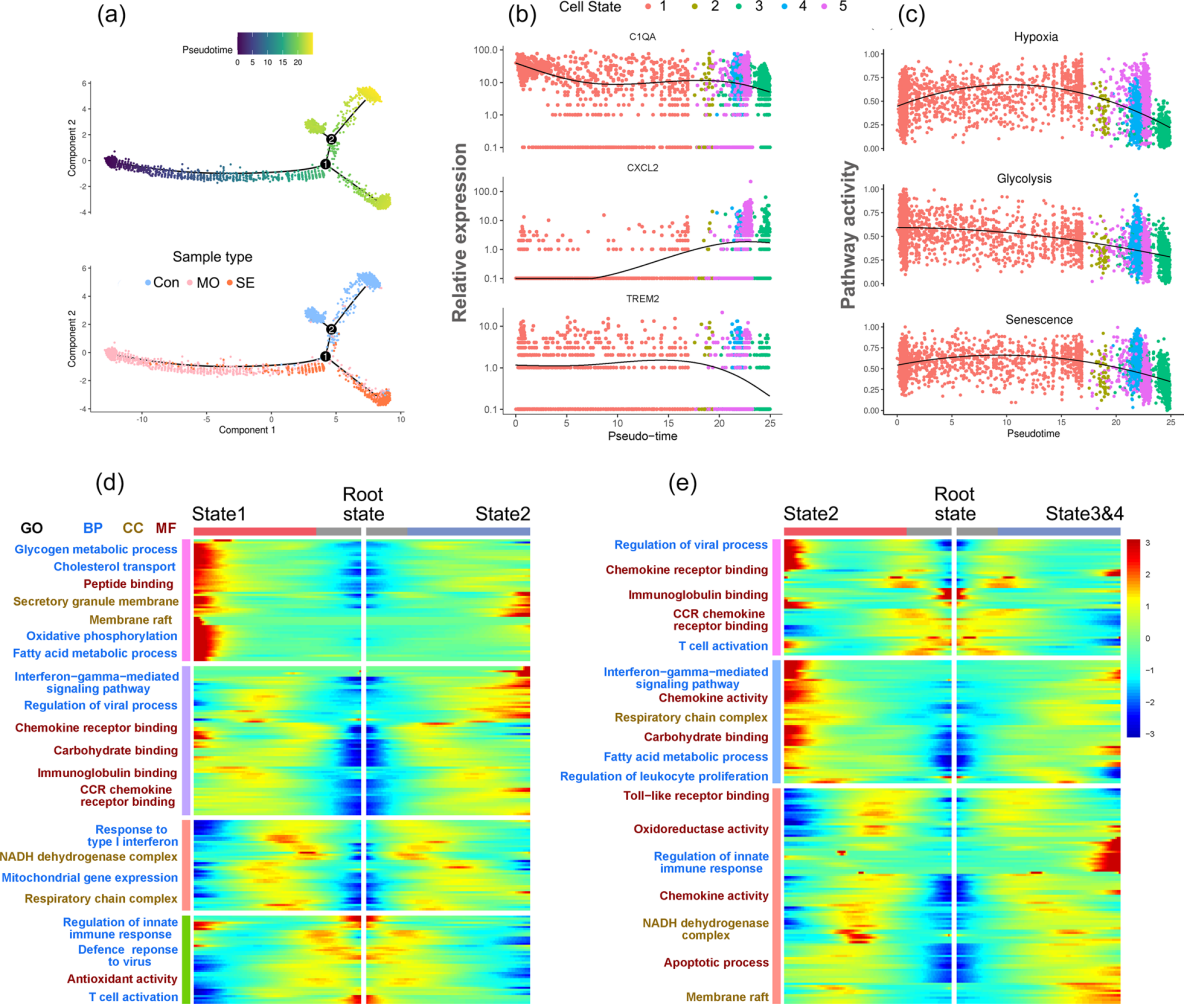
Reconstruction of a trajectory with Macrophage Subpopulation. **a** The single-cell trajectory reconstructed by Monocle contains five main branches and two decision points. Cells are colored based on pseudotime (upper) and sample types. Abbreviation: Con, MO, SE indicate Control, Moderate, and Severe, respectively. **b** Dot plot showing the variability of gene expressions, such as C1QA (upper), CXCL2 (middle), and TREM2, following pseudotime based on cell states. A natural spline was used to model gene expression as a smooth, nonlinear function over pseudotime. **c** Dot plot showing the variability of pathway activity, such as hypoxia (upper), glycolysis (middle), and senescence, following pseudotime in the path that contains cells of states 1, 2, 3, 4, and 5. **d**, **e** Each heatmap presents genes differentially expressed between two branch comparisons, and each row represents the expression level of a gene along the branch trajectory. Enriched pathways are summarized for each gene cluster. From root to state 1 and state 2 branches (**d**), from root to state 2, and combined state 3 with 4 branches (**e**)

Although cell clustering is useful for identifying subtypes, reconstructing cell states in continuous processes is difficult. Pseudo-time trajectory analysis was performed to infer lineage relationships among the macrophage subsets. The trajectory constitutes two decision points and five states, which were derived to capture different sample groups in an orderly manner (Fig. 6a, Additional file 2: Figure S3). Based on the above findings, we considered that the cells traveled from state 1 through branch point 1, state 2, and then to branch point 2, representing clinical outcome progression of the disease, from early infection with SARS-CoV-2 to severe or recovery, during which the expression of representative marker genes including C1QA, CXCL2, and TREM2 in macrophages was changed following pseudo-time (Fig. 6b).

Next, we calculated the cell-specific pathway activity, which may reflect the functional status of the cell trajectory. The trend of the hypoxic signal first increased and then decreased with the pseudo-time (Fig. 6c). Along with this trend, the glycolytic activity gradually decreased, suggesting that cellular glycolysis metabolism was dependent on hypoxia status. Similarly, with fluctuating hypoxia, the senescence status within the cell trajectory was changed accordingly, which is consistent with previous studies involving the regulation of microenvironment and the increased production of mitochondrial reactive oxygen species [28, 29]. To further explore the functional enrichment of the cell states, we next identified DEGs with branch-dependent expression (FDR < 0.01) for branch points 1 and 2. GO analysis revealed that cells traveling from state 1 to state 2 exhibited highly expressed genes involved in glycogen, fatty acid metabolic process, and oxidative phosphorylation, which was in accordance with their sample types derived from severe and moderate cases (Fig. 6d). Notably, the genes were enriched in chemokine receptor binding and regulation of innate immune response pathways when cells traveled to state 3 and 4 from state 2 (Fig. 6e). These results suggested that macrophages may undergo glucose and fatty acid metabolic reprograming in the acute infectious stage, and then trigger signal transduction involving the regulation of immune responses.

### Assessing cell–cell interactions occurring in patients with COVID-19

**Fig. 7:**
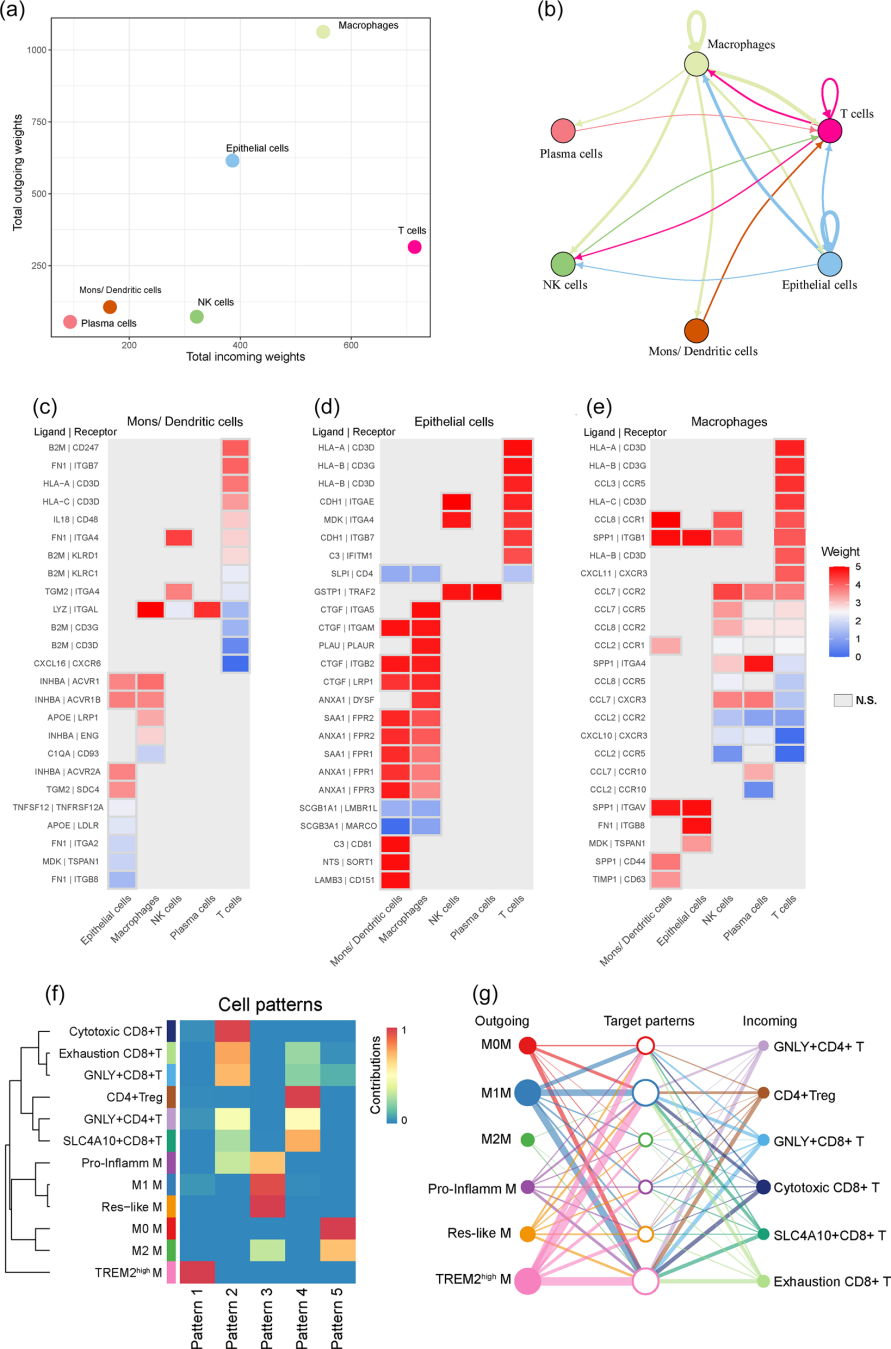
Cell–cell ligand–receptor network analysis. **a** Comparison of total incoming path weights vs total outgoing path weights across BAI cell populations. **b** Circle network diagram of significant cell–cell interaction pathways. Arrows and edge color indicate direction (ligand: receptor) and edge thickness indicates the sum of weighted paths between populations. **c**–**e** Heatmap showing the interaction weights calculated as the product of the average ligand expression from the source cell type including monocyte/ DC (**c**), epithelial cell (**d**) macrophages (**e**), to the average receptor expression of the target cell types. Grey boxes indicate interactions that are not significantly present across all cell types (one-sided Wilcoxon rank-sum test and Benjamini Hochberg false discovery rate [FDR] > 0.05). **f** Heatmap showing the global five communication patterns calculated by the key signals for subpopulations between macrophage and T cells. **g** Hierarchical network diagram of significant cell-cell communication patterns. Edge thickness indicates the sum of weight key signals between populations (from outgoing to incoming)

To identify potential cell–cell interactions that are conserved across the progression of COVID-19, we modeled cases in which both members of a given ligand–receptor interaction are expressed by cell types present within the BALF immune microenvironment. Based on permutation testing of randomized network connections that were constructed with weighted edges reflecting expression fold changes of ligands and receptors in source and target populations as previously described [30], macrophages exhibited the highest number of outbound connections, with having the greatest weight. Accordingly, T cells showed the largest number and weighting of significant inbound connections (Fig. 7a, b). Next, we scored interactions by calculating the average expression of receptors and ligands in the respective cell types mentioned above. We assessed the statistical significance of each interaction score using the Wilcoxon rank-sum test and performed Benjamini–Hochberg multiple hypothesis correction. Compared to Mons/DCs and EPC, NK, and plasma cells, many of the high-scoring interactions were detected with the part of the chemokine family in macrophages-related cell crosstalk, especially when communicating with T cells (Fig. 7c–e, Additional file 2: Figure S4a and b). Chemokine interactions involved in T cell perturbations in COVID-19, including CCR and CXCR, were verified in a recently published report [31].

To further explore the detailed interactions involving macrophage and T cell subpopulations, we first annotated T cell subtypes with markers, such as cytotoxic CD8^+^ T cells upregulated with CD8A, CD8B, and GZMK. In addition, CTLA4, PD-1, and TIGIT were overexpressed in exhausted CD8^+^ T cells (Additional file 2: Figure S5). Next, we further inferred all possible communications in cell sub-populations with CellChat [19]. Accordingly, 45 cell–cell relationships with weighted paths higher than expected by chance (p adj < 0.01) were identified. Hierarchical clustering indicated that the sub-populations of macrophages and T cells were completely separated into different groups, similar to the five interactional patterns (Fig. 7f, g, Additional file 2: Figure S4c).

**Fig. 8:**
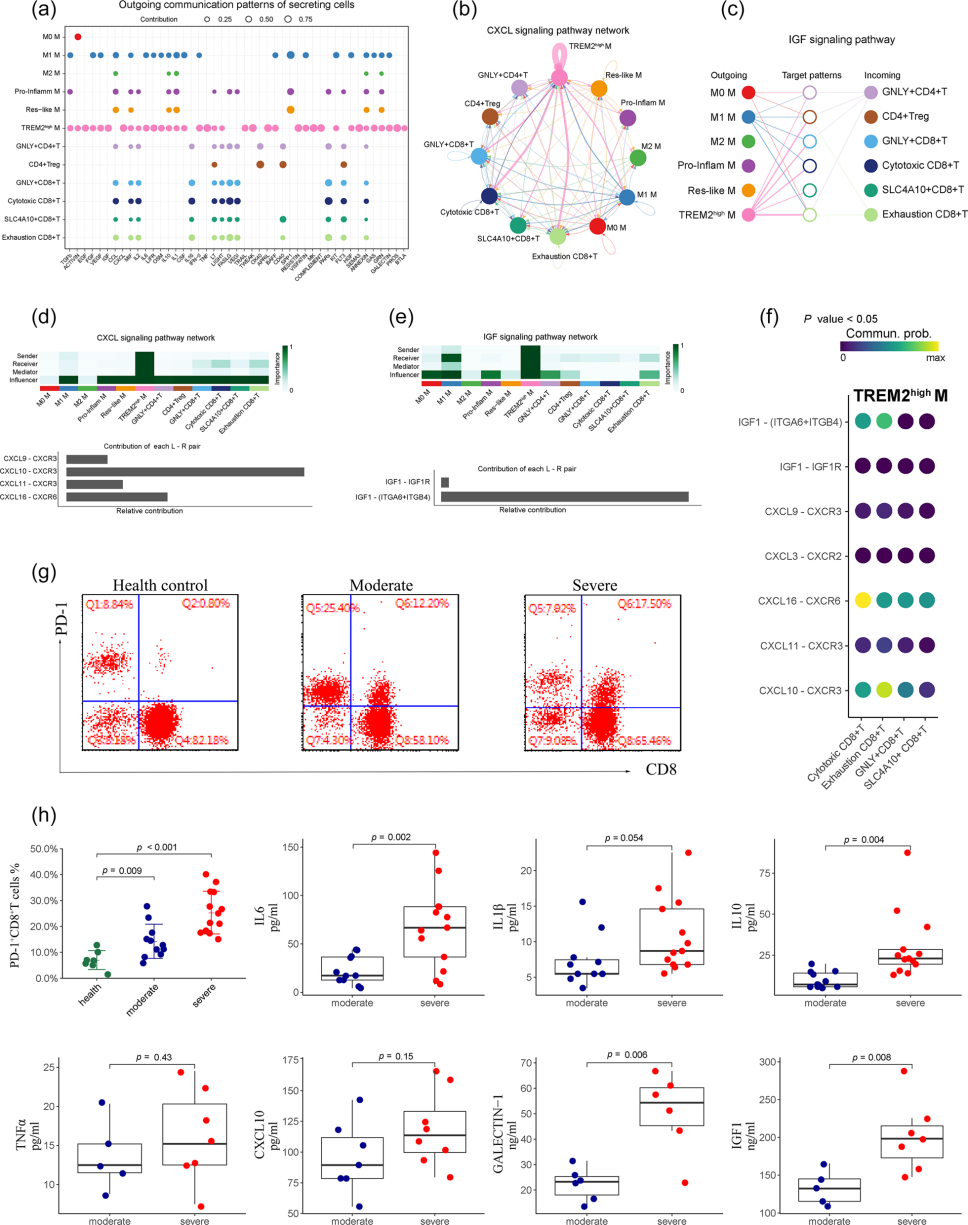
The key communications between macrophage (M) and T cell subpopulation. **a** The dot plot showing the key ligands in the outgoing signaling pattern of subpopulation as secreting cells. **b** Circle plot showing the inferred intercellular communication network for CXCL signaling. Arrows and edge color indicate direction ((source: target). Edge thickness indicates the sum of weight key signals between populations. **c** Hierarchical network diagram of significantly inferred intercellular communication network for IGF signaling based on above-mentioned cell-cell communication pattern (from macrophage to T cell). **d** Heatmap shows the relative importance of each cell subtype based on the computed four network centrality measures of CXCL signaling (upper) and relative contribution of each ligand–receptor pair to the overall communication network of CXCL signaling. **e** Heatmap shows the relative importance of each cell subtype based on the computed four network centrality measures of IGF signaling and the relative contribution of each ligand–receptor pair to the overall communication network of IGF signaling. **f** Significant ligand–receptor pairs involving CXCL and IGF pathway sending signals from TREM2^high^ M to four T cell states. **g** FACS dot plots showing the expression of PD-1 on CD8^+^ T cells. **h** The percentages of PD-1^+^ in CD8^+^ T cells (the first panel). The serum levels of IL6, IL1β, IL-10, TNF-α, CXCL10, GALECTIN-1, and IGF1 in different groups are shown in boxplot (mean ± SD). Statistical analysis by Student’s t-test

We next wanted to address whether observing a ligand–receptor pair correlated with functionally exhausted CD8^+^ T cells during progression after SARS-CoV-2 infection. Firstly, we studied the detailed changes in the outgoing signaling using pattern recognition analysis. We found that TREM2^high^ macrophages dominated the major outgoing signaling, whereas M0 captured the minor component (Fig. 8a). In contrast, at the incoming end of signaling, T cell subpopulations are driven by communication patterns involving pathways including IGF and CXCL (Fig. 8b, c). In particular, CXCL10 and IGF1 derived from TREM2^high^ macrophages were the dominant contributors to this crosstalk (Fig. 8d–f), which is consistent with the previous researches indicating that IGF1 and CXCL signaling influences T cell differentiation [32, 33]. In addition, to quantify the similarity between all significant signaling pathways, we grouped them based on their cellular communication network similarity (Additional file 2: Figure S4d). For instance, IL10 and GALECTIN-1 displayed analogous structure or function with the IGF pathway, suggesting that crosstalk from TREM2^high^ macrophage to T cell subtypes may be mediated by these patterns.

Finally, to further validate the impact of the above-mentioned cytokines on functional exhaustion of CD8^+^ T cells in vivo, we first analyzed the expression of marker related to exhausted T cell (such as PD-1) from 24 patients and seven controls. The expression of PD-1 by CD8^+^ T cells was significantly increased in patients compared to that in control group (Fig. 8g), whereas no significant difference was found between the moderate and severe groups (Fig. 8h). In particular, the levels of IL6, IL10, GALECTIN-1, and IGF1 in severe patients were significantly higher than those in moderate cases, however, there were no differences of IL1β, TNFα, and CXCL10 within the progression of the illness. These data suggested that the exhaustion of CD8^+^ T cells together with several cytokines including IL6, IL10, GALECTIN-1, and IGF1, was associated with the pathogenesis of severe SARS-CoV-2 infection.

## Discussion

In the present study, we first conducted a retrospective analysis of 102 patients with COVID-19, the clinical laboratory findings of which were collected to build a model to support patient diagnosis. We found significant increases in neutrophil%, NLR, AST, D-dimer, and BUN, and decreased platelet counts, lymphocyte %, and lymphocyte count in the severe group (p < 0.01). This result was consistent with accumulated evidence, which was focused on these peripheral blood inflammatory damage parameters for assessing disease severity [1, 21]. For example, the increase in AST, D-dimer, CRP, and BUN abundance indicated a higher level of systemic inflammation and multiple organ damage risks in severe cases than in moderate ones, whereas the inverse proportion of WBC and lymphocyte counts also suggested the disorders involving the immune system [34, 35]. In line with this, our study again confirmed for the predictive models containing the above-mentioned inflammatory damage parameters, with AUROCs ranging from 0.78 to 0.87.

Next, to further explain whether uncontrolled inflammatory innate responses and impaired adaptive immune responses are responsible for the severity of COVID-19, we took advantage of the published scRNA-seq data, derived from the BALF samples of the lower respiratory tract of COVID-19 patients [12] to provide a detailed analysis of intercellular communications. First, when comparing between patients, immune cell clusters were highly patient-specific. T cells mostly consisted of the cells from moderate patients, whereas the proportion of Mons/ DCs in COVID-19 patients was very low. Overall, the macrophages accounted for the highest proportion in all cell clusters, and no significant bias was observed in macrophages from patients. This result is in agreement with other studies, which showed that macrophages are the most enriched immune cell types in the lungs of COVID-19 patients and play a major role in the dysregulated innate immune responses with exaggerated inflammatory cytokine production [3, 4].

Macrophages are morphologically and phenotypically diverse cells [36], which promoted us to presume the existence of cells with high inflammatory potential, combining the crucial role in mediating acute immune responses that cause pathological tissue damage and T cell dysfunction in patients with severe disease. We found that a group of macrophages contributed by COVID-19 cases showed higher hypoxia scores, and there seems to be a mutually collaborative pattern between metabolic activity and inflammatory signaling pathways, including glycolysis, fatty acid biosynthesis, tyrosine metabolism, cytokine receptor interaction, and NF-κB signaling. Macrophages with high metabolic activity tend to have inflammatory potential, which is supported by the recent reports that remodeled metabolism is known to be vital for a macrophage-mediated inflammatory responses [37–39]. Next, we introduced the pseudotime method to capture and dissect transcriptional changes in cells along with the disease progression. Pseudotime analysis ordered the macrophages into five states with a branched structures. One branch represents the root of the trajectory, which showed relatively high C1QA expression, indicating an early stage of acute inflammation. Branch-dependent expression analysis also revealed that many known adaptive immune response-associated genes, such as CXCL2 and IL10, exhibited a gradual increase in expression. Importantly, a continuous and heterogeneous process involving hypoxic status, glycolysis, and senescence pathway activity declined along the trajectory. Notably, TREM2^high^ macrophages were enriched to the root and state1 of trajectory, and could contribute to a major viral infection-induced immune signaling hub. TREM2 activation in macrophages has been shown to lead to the adjustments in their phenotype in response to changing conditions in tissues, including cell maturation under physiological conditions and phenotypic transformation of macrophages upon inflammation [40, 41]. Moreover, cytokine storm is a serious life-threatening condition characterized by uncontrolled activation of macrophages, which accumulate in the lungs and are the likely source of pro-inflammatory cytokines and chemokines, including IL6, IL1β, and CXCL8 [42]. In addition, macrophages can regulate T cell inhibition through direct cell-to-cell contact as well as through the secretion of cytokines and metabolic byproducts [43]. For example, the TREM2^high^ macrophage population which has an activated complement system could be the source or consequence of complement activation contributing to the blockade of CD8^+^ T cell activation [44].

In accord with this, a recent report has demonstrated that the abundance and interactions within epithelium-immune cells at the single-cell level are predictive of COVID-19 severity [45]. Accordingly, we also used correlative and predictive models to identify cell–cell interactions involving subtypes of macrophages and T cells that may be related to the clinical features of functional exhaustion of T cells in the disease. Based on the ligand–receptor (L-R) enrichment analysis, macrophages and T cells could be segregated into 12 major sub-populations, among which, TREM2^high^ macrophages showed a distinct secretory (outgoing) link to CD8^+^ T cells, highlighting the likely functional communication between them. Indeed, the similarity of signaling network topological analysis inferred that a subtype of macrophages may be the primary ligand source, which acts in a paracrine manner to CD8^+^ T cells, especially exhaustion CD8^+^ T cells. Notably, CXCL10, IGF1, and GALECTIN-1 were the dominant contributors to this communication signaling, which is supported by previous reports that cytokines, such as GALECTIN-1, mediate immune evasion by preventing T cell migration, and blocking CD8^+^ T cell activation [25, 46, 47].

In line with this, we also observed gradually elevated levels of serum proinflammatory cytokines, including IL6, IL1β, CXCL10, and IGF1, which are known to contribute to the increased severity of disease caused by certain strains of coronavirus. In addition, a significant increase in suppressive cytokines, such as IL10 plays a vital role in limiting excessive inflammation, the results of which suggest that the balance between cytokines involving immunoactivation and immunosuppression may be associated with the pathogenesis of SARS-CoV-2 infection.

We are aware of three limitations of our study. Firstly, the number of patients in this study is relatively small. The results should be validated in another prospective study. Second, due to fact that technical limitation, the experiments involving animal or cellular model were not performed to verify the interaction of macrophage and T cell after SARS-Cov-2 infection, the direct evidence of which should be further elucidated in assays. Third, we only analyzed peripheral blood lymphocytes. A further analysis of lymphocytes from alveolar lavage fluid is needed.

Taken together, our study used scRNA-seq to identify a subtle communication between BAI sub-populations during COVID-19 progression, which revealed that TREM2^high^ macrophages driving ligand–receptor crosstalk at a high resolution contributed to the exhaustion of CD8^+^ T cells. These new insights into COVID-19 progression may be useful for a better understanding of the differences in the clinical symptoms after SARS-CoV-2 infection, and the identified serum cytokine panel, including IL6, IL1β, IL10, CXCL10, IGF1, and GALECTIN-1, may represent a selective targets to improve clinical therapeutic effects.

## Conclusions

### Decreased counts of total T Cells, CD4^+^, and CD8^+^ subsets in COVID-19 patients with severity

Currently, the world is experiencing a severe new pandemic, health workers have gone the extra mile to fight against COVID-19. In this paper, we focus on the potential molecular mechanism of clinical severity-dependent reduction in T cell numbers, which is the hallmark of severe COVID-19 cases. Firstly, we found the number of T cells such as total T cells, CD4 ^+^ and CD8 ^+^ T cells in the severe disease group were significantly lower than in the moderate disease group. Furthermore, the ROC curve indicates the absolute numbers of total T cells and CD8 ^+^ T cells are the good factors for the prediction of COVID-19 severity. There is an urgent need for foundational information about T cell responses to this virus because such knowledge can guide the selection of vaccine strategies most likely to elicit protective immunity against SARS-CoV-2.

### Single-cell RNA-seq reveals the increased clonal expansion of macrophages was found in severe COVID-19 cases

To further elucidate the molecular mechanism of above-mentioned clinical features, we analyses transcriptome profiles at the single cell level, which was one of the first available datasets to demonstrate the landscape of BALF immune cells in patients with COVID-19.

It is known that inflammatory macrophages played a crucial role in phagocytosis and eliminating bacterial, uncontrolled activation of inflammatory macrophages my trigged systemic inflammatory response syndrome. Importantly, remodeled metabolism is known to be vital for a macrophage-mediated inflammatory response. In line with this, we detected whether macrophages heterogeneity was responded to SARS-CoV-2 infection. We indeed found a group of macrophages, which upregulated TREM2 expression level showed higher hypoxia score, importantly, there seem to be mutually collaborative pattern between metabolic activity and inflammatory signaling pathway, including glycolysis, fatty acid biosynthesis, tyrosine metabolism, cytokine receptor interaction and NF-κB signaling. Therefore, precise regulation of TREM2^high^ macrophages is of paramount importance in guaranteeing microbial clearance, injury limitation, and avoidance of serious side effects.

### Cell communication analysis based on ligand–receptor pattern could provide clues to explain reduction and exhaustion of CD8^+^ T cells in COVID-19

Using the scRNA-seq data derived from BALF samples, we demonstrated that macrophages and T cells could be segregated into 12 major sub-populations, among which, TREM2^high^ macrophages showed a distinct secretory (outgoing) link to exhaustion CD8^+^ T cells, highlighting the likely functional communications between them. To further validate the findings, as disease severity progresses in patients with COVID-19, we detected a concomitant rise in serum cytokine levels associating immune cell communication, such as IL6, IL10, IGF1, and GALECTIN-1, may play roles on reduction and exhaustion of T cell populations.

In conclusion, the ability to examine cell type-specific communication provided by scRNA-seq enables a broad range of applications, which identify interactions that are predictive biomarkers of response to therapy for use in patient with COVID-19.

## Supplementary Information

**Additional file 1.** **Table S1.** Univariate and multivariate logistic regression of clinical characteristics. 

**Additional file 2.** **Supplemental Figures S1-S4.** The analysis of scRNA-seq data related to main figures.

## Data Availability

The raw data for scRNA-seq analysis in this study were downloaded from GEO database (GSE145926). These data were published by Liao et al. [12], which extracted BALF cells from nine COVID-19 patients and three healthy control. The codes related to bioinformatics analysis are available from the corresponding author on reasonable request.

## Abbreviations

COVID-19: Coronavirus disease 2019
ScRNA-seq: Single cell RNA-sequencing
SARS-Cov-2: Severe acute respiratory syndrome coronavirus 2
ARDS: Acute respiratory distress syndrome
SIRS: Systemic inflammatory response syndrome
BALF: Bronchoalveolar lavage fluid
WBC: White blood cell
RBC: Red blood cell count
NLR: Neutrophil to Lymphocyte ratio
HB: Hemoglobin
PLT: Platelet count
ALT: Alanine aminotransferase
AST: Aspartate aminotransferase
TBIL: Total bilirubin
BUN: Blood urea nitrogen
CRP: C-reactive protein
PT: Prothrombin time
APTT: Activated partial thromboplastin time
Cr: Creatinine
CK: Creatine kinase
PCT: Procalcitonin
BNP: Brain natriuretic peptide
FIB: Fibrinogen
OR: Odds ratio
AUROC: Area under the receiver operating characteristic curve criterion
BAI cells: Bronchoalveolar lavage fluid (BALF) immune cells
MACs: Macrophages
Mon/ DCs: Monocytes/dendritic cells
NKCs: Natural kill cells
PMCs: Plasma cells
ECs: Epithelial cells
DPA: Permutation-based differential proportion analysis
GO: Gene Ontology
GSEA: Gene Set Enrichment Analysis
GSVA: Gene Set Variation Analysis
DEGs: Differentially expressed genes
SD: Standard deviations
FDR: False discovery rate
