# Supplementary material for: Single-cell analysis reveals cell communication triggered by macrophages associated with the reduction and exhaustion of CD8+ T cells in COVID-19
Source: Cell Commun Signal. 2021 Jul 8;19:73. doi: 10.1186/s12964-021-00754-7 (PMC8264994; doi:10.1186/s12964-021-00754-7)
Supplement: Supplementary file 2 — Additional file 1. Table S1. Univariate and multivariate logistic regression of clinical characteristics. [file 12964_2021_754_MOESM2_ESM.pdf]

Table S1. Univariate and multivariate logistic regression

Characteristic	Univariate analysis		Multivariate analysis	
	**OR (95% CI)**	**P value**	**OR (95% CI)**	**P value**
**Age**	0.29(0.12 - 0.65)	0.003**		
**Gender**	1.12(0.54 - 1.7)	0.650		
**WBC**	1.16(1.05 - 1.3)	0.009**	0.82(0.67 – 1.02)	0.071
**Neu%**	1.08(1.04 - 1.13)	< 0.001***		
**Lymph%**	0.94(0.9 - 0.98)	0.007**		
**RBC**	1.12(0.63 - 1.67)	0.700		
**HB**	0.99(0.96 - 1.05)	0.744		
**PLT**	0.91(0.85 - 0.98)	0.009**		
**ALT**	0.97(0.94 - 1.02)	0.058		
**AST**	1.04(1.02 - 1.07)	< 0.001***	1.04(1.01 - 1.07)	0.008
**TBIL**	1.07(1.02 - 1.13)	0.018*		
**BUN**	1.23(1.09 - 1.43)	0.004***	1.12(0.99 - 1.27)	0.041
**Cr**	1.06(0.95 - 1.09)	0.222		
**CK**	1.02(0.91 - 1.05)	0.058		
**CRP**	1.01(0.92 - 1.03)	0.060		
**PCT**	1.04(0.95 - 1.13)	0.146		
**BNP**	1.05(0.93 - 1.07)	0.255		
**PT**	1.41(1.1 - 1.85)	0.009**		
**APTT**	1.04(0.94 - 1.16)	0.449		
**D-dimer**	1.23(1.1 - 1.47)	0.006**	1.23(1.013 - 1.50)	0.037
**FIB**	1.17(0.85 - 1.64)	0.342		
