# Supplementary material for: Single-cell analysis reveals cell communication triggered by macrophages associated with the reduction and exhaustion of CD8+ T cells in COVID-19
Source: Cell Commun Signal. 2021 Jul 8;19:73. doi: 10.1186/s12964-021-00754-7 (PMC8264994; doi:10.1186/s12964-021-00754-7)
Supplement: Supplementary file 3 — Additional file 2. Supplemental Figures S1-S4. The analysis of scRNA-seq data related to main figures. [file 12964_2021_754_MOESM3_ESM.pdf]

Supplementary Figures

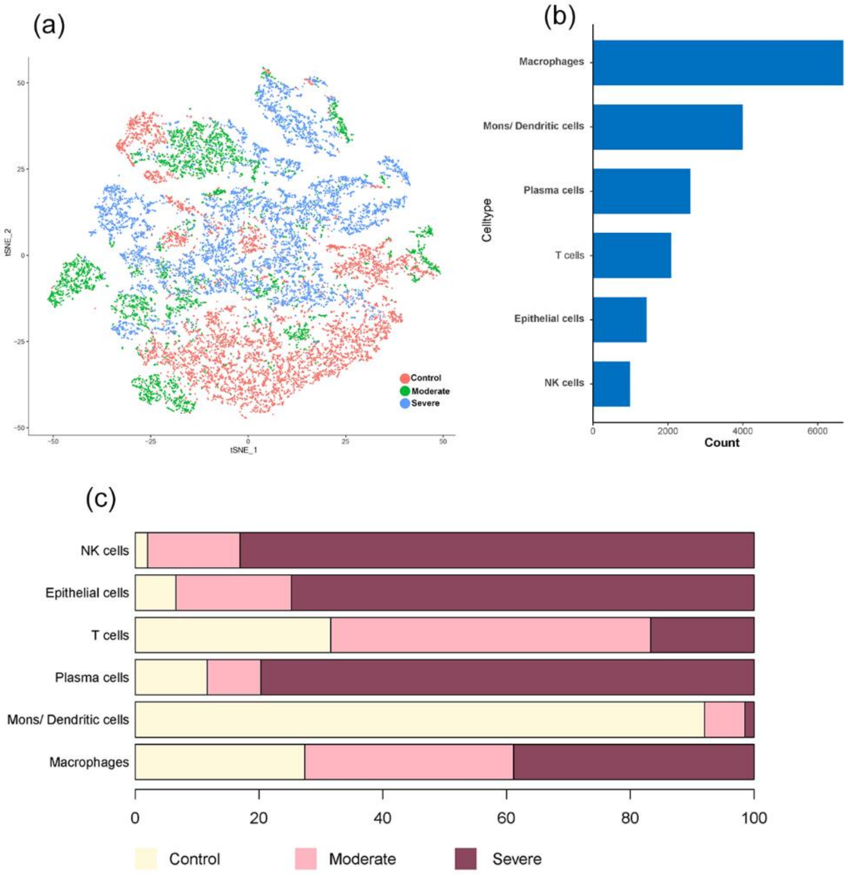

Figure S1. The cell subtypes were identified by scRNA-seq derived from Liao *et al.* (2020).

(a) t-SNE plot showing the distribution of the patients matching.

(b) The numbers of BALF immune cell subtypes.

(c) The fraction of cells originating from each of three types of sample including healthy control, the patients with moderate and severe COVID-19.

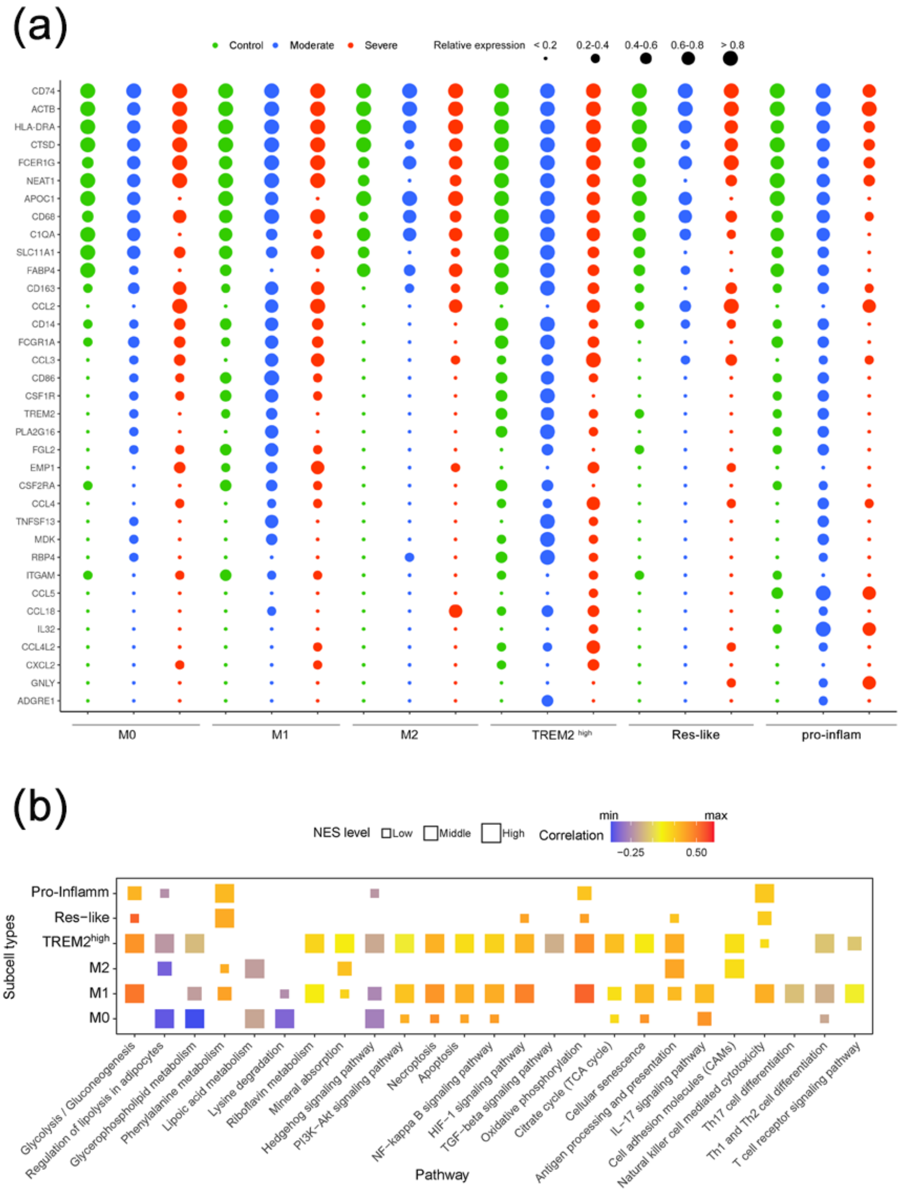

Figure S2. Hypoxia status of each macrophage subpopulation.

(a) Dot-plot of top marker genes for each macrophage population where color indicates different conditions with COVID-19 patients.

(b) Summarized correlation of hypoxia status and signal pathway activity across macrophage subpopulations.

The blank in plot means the correlation is not statically significant.

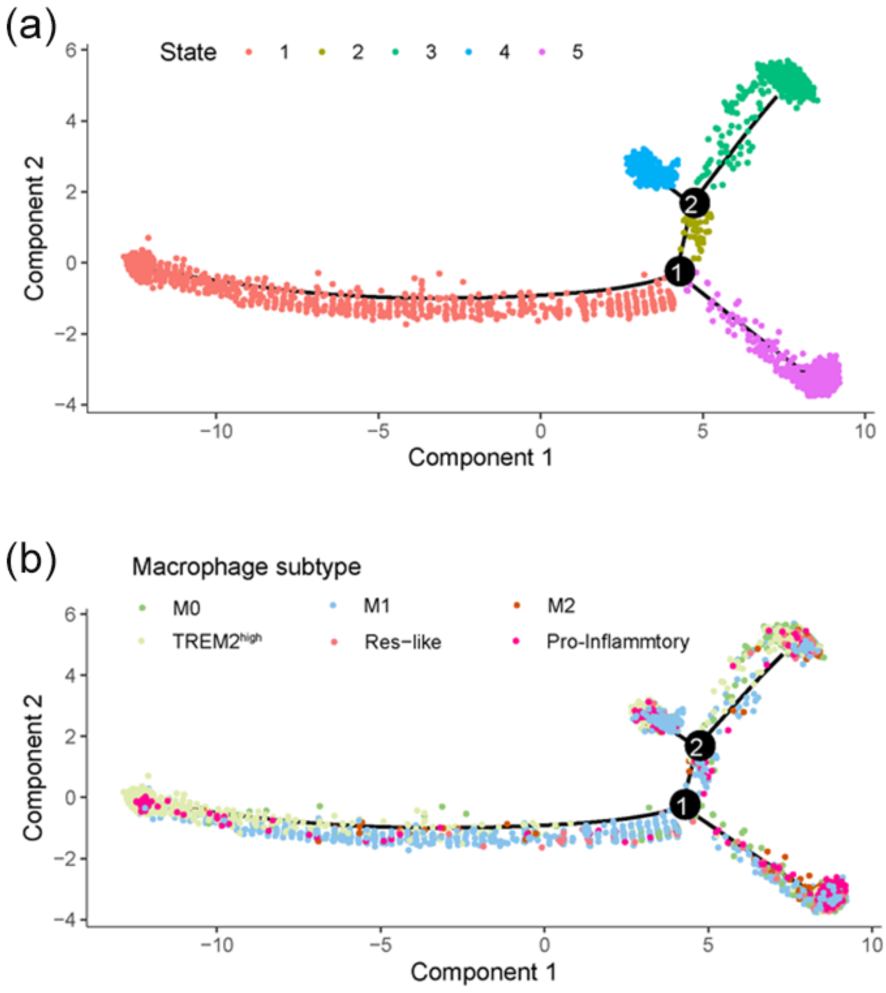

Figure S3. The single cell trajectory reconstructed by Monocle contains five main branches and two decision points. Cells are colored based on trajectory states (a) and macrophage subtypes (b).

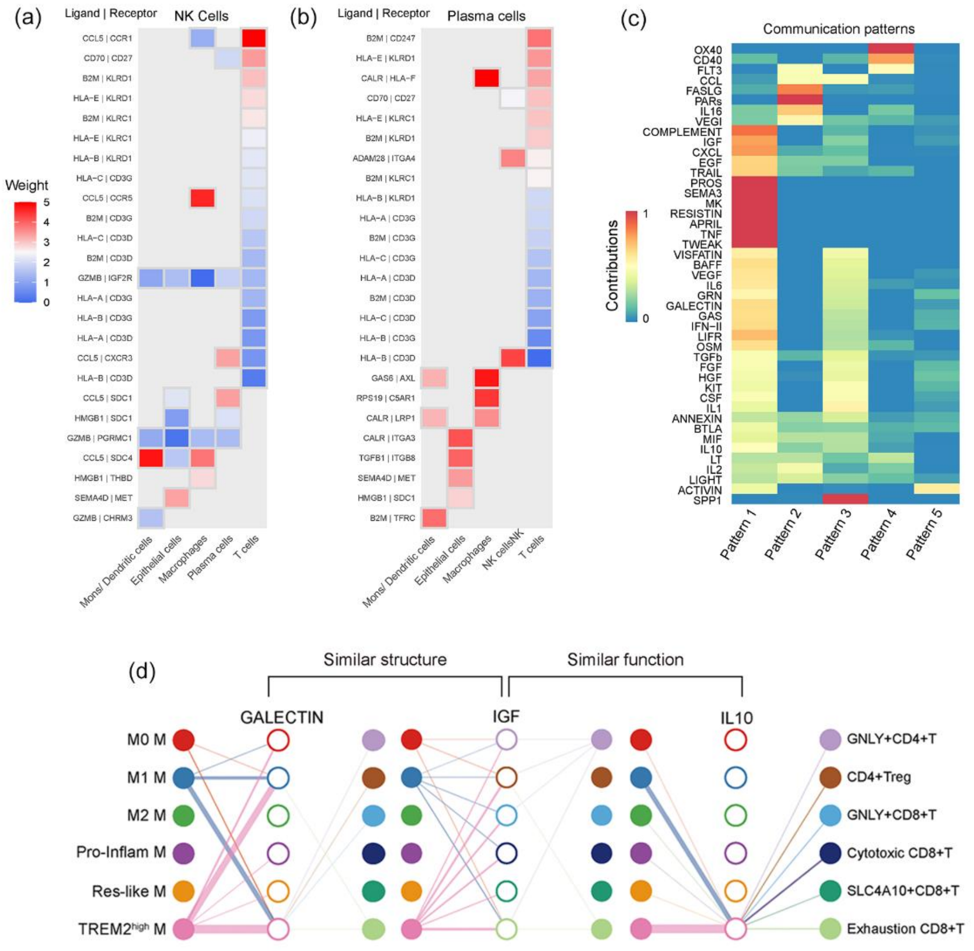

Figure S4. Cell communication analysis between macrophage and T cells.

(a-b) Heatmap showing the interaction weights calculated as the product of the average ligand expression from the source cell type including NK cells (a) and plasma cells (b), to the average receptor expression of the target cell types. Grey boxes indicate interactions that are not significantly present across all cell types (one-sided Wilcoxon rank-sum test and Benjamini Hochberg false discovery rate [FDR] > 0.05).

(c) Heatmap showing the global five communication patterns calculated by the marker genes from macrophage to T cells.

(d) Hierarchical network diagram of significant cell-cell communication patterns based on analogical structure or function analysis. Edge thickness indicates the sum of weight key signals between populations (from outgoing to incoming).

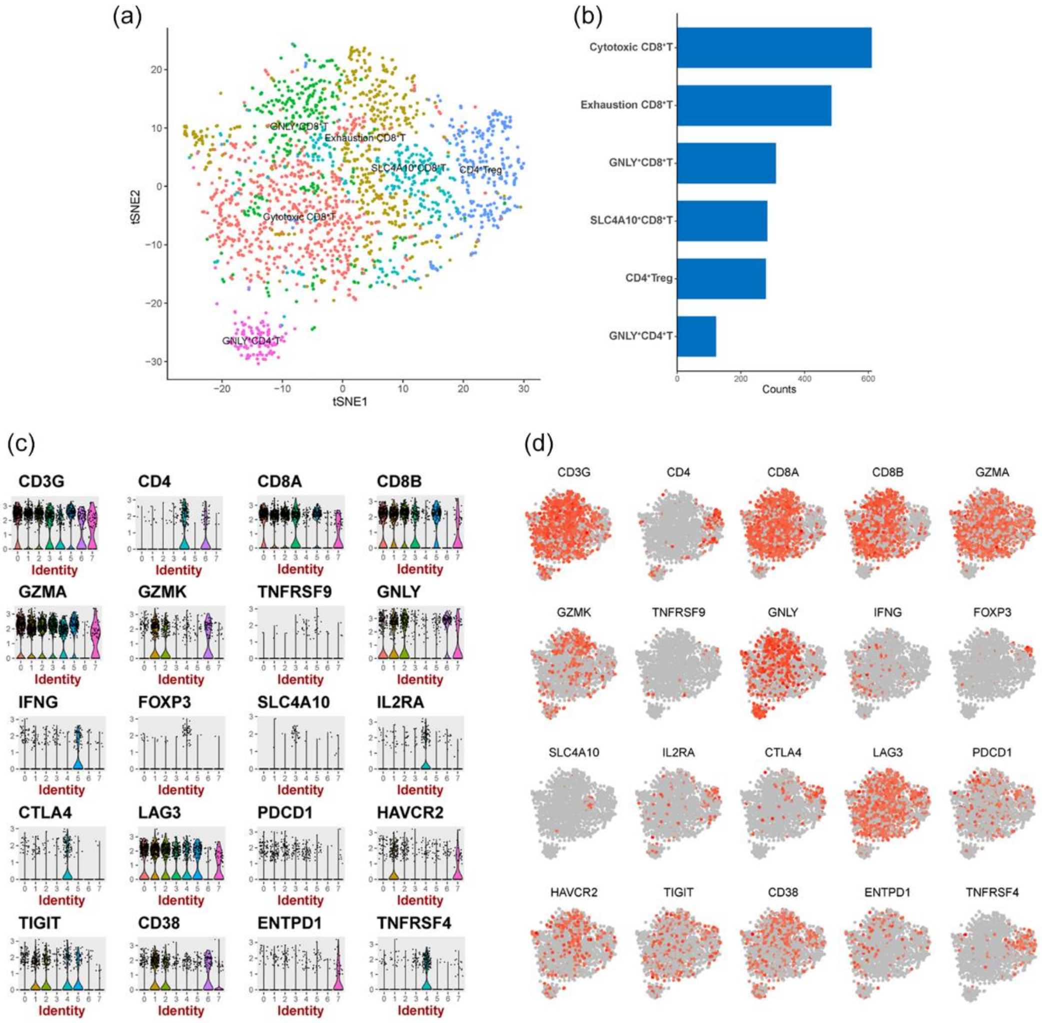

Figure S5. T cell subtypes were identified by scRNA-seq analysis.

(a) t-SNE plot showing T cell populations.

(b) The cell numbers of each subpopulation of T cells.

(c) Violin plots showing marker genes across identities.

(d) Expression of marker genes across T cell subtypes as visualized on t-SNE.
